# Creating a Superior *Wx* Allele with Temperature-Responsive Amylose Regulation and a Novel Transcriptional Pattern in Rice via CRISPR/Cas9-Mediated Promoter Editing

**DOI:** 10.3390/foods14081330

**Published:** 2025-04-11

**Authors:** Jiali Yan, Jiawen Yu, Huimin Shen, Lihui Zhou, Zhuanzhuan Chen, Xiaolei Fan, Qianfeng Li, Changquan Zhang, Qing Liu, Lichun Huang, Qiaoquan Liu

**Affiliations:** 1 Jiangsu Key Laboratory of Crop Genomics and Molecular Breeding, Zhongshan Biological Breeding Laboratory, Key Laboratory of Plant Functional Genomics of the Ministry of Education, Agricultural College of Yangzhou University, Yangzhou 225009, China; 2 Jiangsu Key Laboratory of Crop Genetics and Physiology, Co-Innovation Center for Modern Production Technology of Grain Crops of Jiangsu Province, Yangzhou University, Yangzhou 225009, China; 3 Jiangsu High Quality Rice Research and Development Center, Jiangsu Key Laboratory for Agro-Biology, Institute of Food Crops, Jiangsu Academy of Agricultural Sciences, Nanjing 210014, China; 4 Yangzhou Modern Seed Innovation Institute, Gaoyou 225600, China

**Keywords:** *Wx* gene, amylose content, grain transparency, promoter editing, transcriptional pattern

## Abstract

High quality stands as a pivotal competitive edge in the rice industry. Optimizing amylose content (AC) and the physicochemical properties of endosperm starch by regulating the *Wx* gene is crucial for enhancing rice grain quality. In this study, we created a novel *Wx^b-d25^* allele by deleting a 25 bp segment (−26 to −2) within the *Wx* core promoter using CRISPR/Cas9. Compared with the wild type and the previously reported *Wx^b-i1^*, *Wx^b-d25^* exhibited no significant changes in agronomic traits. However, its grains displayed temperature-dependent variations in AC and altered transparency and viscosity characteristics, holding the potential to synergistically improve both the eating and cooking quality (ECQ) and appearance quality (AQ) of rice. Further studies demonstrated that this promoter modification, by partially disrupting the transcription initiator, significantly downregulated the original *Wx*-01 transcript and generated a novel *Wx* transcript (ONT.7395.1) in *Wx^b-d25^* grains. Despite its low expression abundance, the ONT.7395.1 transcript could be completely processed into mature *Wx* mRNA. The combined effects of the dual transcripts resulted in significantly increased *Wx* gene expression and AC in *Wx^b-d25^* grains under conventional cultivation conditions. These findings provide a genetic resource and a theoretical foundation for utilizing the *Wx^b-d25^* allele to improve rice grain quality.

## 1. Introduction

High-quality rice is a central objective in genetic improvement programs, with starch composition and structure playing a decisive role in overall grain quality [1,2]. Among starch-related characteristics, the amylose content (AC), which reflects the ratio of amylose to amylopectin in the endosperm, is the primary determinant of rice physicochemical properties [3,4,5]. Research has shown that AC profoundly influences eating and cooking quality (ECQ), appearance quality (AQ), nutrition quality (NQ), and milling quality (MQ) [5,6,7,8,9]. For ECQ, AC correlates positively with rice hardness and inversely with stickiness [10]. Modulating AC of rice exerts a significant influence on its physicochemical attributes, including hardness and stickiness, which in turn significantly enhances the palatability of cooked rice [3]. Globally, consumer preferences for rice with varying AC levels are highly diverse [11,12]. In East Asian countries such as China and Japan, rice with low AC (typically <16%) enjoys widespread popularity due to its softer texture and higher stickiness [11,12]. Conversely, in regions like India and Pakistan, consumer preference leans toward rice varieties characterized by higher hardness and lower stickiness, which generally correspond to medium-to-high AC levels (exceeding 20%) [11,12]. Furthermore, glutinous rice, which lacks amylose synthesis, has gained widespread application in the processing of rice-based foods, thanks to its distinctive physicochemical attributes [11]. However, its use as a staple food on a long-term basis remains relatively limited. These divergent preferences likely stem from the distinct dietary habits and culinary traditions that prevail in different regions. Concerning AQ, AC correlates positively with grain transparency. When AC is lower than about 12%, transparency declines significantly, rendering the grains increasingly translucent or even opaque [13,14]. In addition, medium- to high-AC varieties are more susceptible to chalkiness, while suppressing amylose synthesis can significantly mitigate chalkiness formation [15,16]. From a nutritional perspective, higher AC promotes resistant starch, thereby reducing the rate of digestion [17,18,19]. As for MQ, AC is intricately linked to both rice grain fissure resistance and head rice yield [20]. Lowering AC significantly boosts head rice yield. In summary, while there are clear differences in consumer preferences for rice AC, global research on rice domestication and artificial selection has consistently revealed a trend toward selecting for lower AC levels [3,21]. However, the degree of AC reduction in rice varies across regions due to differing consumer preferences. Therefore, adjusting rice AC in a region-specific manner to meet consumer preferences—without considering digestive characteristics—emerges as a fundamental strategy for enhancing rice quality.

Amylose synthesis in the rice endosperm is controlled by the *Waxy* (*Wx*) gene, which encodes granule-bound starch synthase I (GBSSI) [22]. The precise genetic regulation of the *Wx* gene and its encoded GBSSI protein represents a critical avenue for rice quality improvement. Multiple regulatory pathways, both direct and indirect, modulate the expression and activity of *Wx*, with transcriptional and post-transcriptional mechanisms playing a key role. For example, a G/T mutation at the first base of the first intron disrupts the processing of pre-mRNA into mature mRNA, accounting for the markedly lower AC observed in rice carrying the *Wx^b^* allele compared to that with *Wx^a^* [23,24]. Surrounding this variation site, several *Dull* genes and quantitative trait loci (QTLs) have been identified that modulate *Wx* splicing efficiency in the *Wx^b^* background, including *Du1*, *Du2*, *Du3*/*OsCBP20*, *LowAC1*, *LAC6*/*TL1*/*Du13*, *qAC2*, and *qSAC3* [25,26,27,28,29,30,31,32]. Recent studies have identified multiple transcription factors involved in *Wx* regulation, including OsbZIP58, OsbZIP33/REB, OsNAC20, OsNAC26, NF-YB1, NF-YC12, bHLH144, OsMADS7, OsMADS14, RSR1, SLRL2/LCG1, OsMYB73, and OsDOF18. These factors regulate AC by either directly binding to the *Wx* promoter or indirectly interacting with other transcription factors that influence *Wx* expression [16,33,34,35,36,37,38,39,40,41,42]. Additionally, DNA methylation has been implicated in the transcriptional regulation of *Wx*. High levels of CpG methylation in the *Wx* promoter are closely associated with reduced AC, with two adjacent CpG islands identified in this region [43]. Promoter editing studies have further refined our understanding of *Wx* gene regulation, uncovering additional expression-modulating elements beyond known transcription factor binding sites. For example, our previous research identified and validated an S7 site within the *Wx* core promoter that effectively fine-tunes *Wx* expression [44]. Similarly, Zeng et al. identified multiple *cis*-acting elements in the *Wx* promoter, including the Endosperm-box, A-box, and CAAT-box [45]. These findings highlight the complexity of *Wx* transcriptional regulation and provide valuable insights into strategies for targeted modulation to enhance rice quality.

The advent and advancement of CRISPR/Cas technology have significantly streamlined research into gene functions and the development of superior alleles and novel germplasms [46]. Presently, CRISPR/Cas technology has been extensively utilized for gene editing in a variety of crops, with the *Wx* gene emerging as one of the most frequently targeted genes. To date, successful editing of the *Wx* gene using CRISPR/Cas technology has been reported in several major crops, including rice, maize, wheat, barley, and cassava [47,48,49,50,51]. These studies have not only expanded the potential for creating superior *Wx* alleles with diverse AC profiles through gene editing but also paved the way for a deeper exploration of the regulatory mechanisms governing the *Wx* gene.

In this study, we focused on Chinese rice consumers who favor low AC. We tackled the challenge that the popular low-AC soft rice varieties (typically <12%) in breeding and consumer markets, while offering good palatability due to their low AC, often suffer from poor grain transparency, thus failing to achieve a balance between excellent ECQ and desirable AQ. By leveraging our previously established method of editing the core promoter of the *Wx* gene to fine-tune its expression and regulate rice AC, we successfully developed a novel *Wx^b-d25^* allele featuring a 25 bp deletion within the *Wx* core promoter. We compared the AC and transparency of rice carrying this new allele with those of the representative soft rice variety Nangeng 46 and conducted a comprehensive evaluation of the physicochemical properties of the relevant materials. Furthermore, we elucidated the underlying mechanism by which this allele modulates *Wx* gene expression and rice AC through alterations in the transcriptional pattern of the *Wx* gene. These findings not only introduce a new gene and germplasm resource but also provide a robust theoretical foundation for improving the quality of soft rice.

## 2. Materials and Methods

### 2.1. Plant Materials

The *japonica* rice variety Nipponbare, which carries the *Wx^b^* allele, served as the recipient for generating the promoter-edited lines. The elite soft rice cultivar Nangeng 46 (NG46), which carries the *Wx^mp^* allele, was used as a control due to its low AC and excellent ECQ [52]. All rice lines were cultivated under identical climatic and management conditions in a paddy field in the summer in Yangzhou, Jiangsu Province (119° E, 32° N) or winter in Lingshui, Hainan Province (110° E, 18° N).

### 2.2. Construction of CRISPR/Cas9 Vector and Screening of Homozygous Edited Lines

The CRISPR/Cas9 system used in this study includes two parts: the intermediate vector *SK-gRNA* and the final vector *pC1300-Cas9* [53]. The target site S7 was designed as a primer and inserted into the gRNA region of *SK-gRNA*. Afterward, the gRNA was cleaved and assembled into the *pC1300-Cas9* binary vector using an isocaudamer system. The final CRISPR/Cas9 construct was transformed into rice via *Agrobacterium*-mediated transformation. To determine mutation type, the targeted promoter region was amplified by PCR and sequenced using the Sanger method. The sequencing data were analyzed via DSDecodeM (http://skl.scau.edu.cn/dsdecode/) (accessed on 2 August 2024) or manually decoded [54,55]. A list of the PCR primers used is provided in Table S1.

### 2.3. Determination of Key Agronomic Traits

Wild-type (WT) and S7-edited rice plants were dug up from the field at 15–25 days after flowering (DAF) and photographed to compare overall plant morphology. Once the rice reached maturity, 10 individual plants were randomly selected to measure plant height and tiller number, while 10 main panicles were sampled for evaluating seed setting rate. After drying, more than 50 rice grains were scanned using an SC-E rice appearance quality detector (Wanshen Testing Technology Co., Hangzhou, Zhejiang, China) to measure grain length, grain width, and 1000-grain weight. Grain thickness was measured on 15–20 grains using a vernier caliper.

### 2.4. Determination of Physicochemical Properties of Rice Grains

Mature seeds were air-dried, dehulled (SY88-TH, Sangyong, Seoul, Republic of Korea), polished (Pearlest, Kett, Tokyo, Japan) and ground into powder (Cyclotec CT1093, FOSS, Alingsås, Sweden), then passed through a 100-mesh sieve (pore size 0.15 mm). Total starch content (TSC) was measured using a K-TSTA total starch assay kit (Megazyme, Bray, County Wicklow, Ireland). The measurement of apparent amylose content (AAC) and gel consistency (GC) was conducted in accordance with previously published methods [56]. Gelatinization temperature (GT) was measured using a 200 F3 differential scanning calorimeter (DSC, Netzsch, Selb, Germany), providing onset (T_o_), peak (T_p_), and end (T_e_) temperatures, along with the enthalpy of gelatinization (ΔH). Pasting properties were assessed using a Techmaster rapid visco-analyzer (RVA) (Newport Scientific, Warriewood, NSW, Australia). The RVA parameters include peak viscosity (PKV), hot paste viscosity (HPV), breakdown value (BDV), cool paste viscosity (CPV), setback value (SBV), peak time (PeT), and pasting temperature (PaT). All measurements were performed in triplicate.

### 2.5. Grain Transparency Calculation

Rice grains from various lines were air-dried, dehulled (SY88-TH, Sangyong, Korea), polished (Pearlest, Kett, Japan), and then placed on a scanner coupled with the SC-E rice appearance quality detector (Wanshen Testing Technology Co.) to obtain high-resolution photos. Using ImageJ 1.53t software, the images were converted to grayscales, inverted, and then measured for grayscale values. Dividing these values by 255 yielded the grain transparency [27,56].

### 2.6. Scanning Electron Microscopy Analysis

Starch was purified from milled rice via a reported neutral protease method [57] with slight modifications. The resulting starch samples were suspended in ethanol, mounted on aluminum stubs using carbon double-sided conductive tape, gold-coated using a sputter coater, and then examined using a Hitachi S-4800II environmental scanning electron microscope (SEM, Hitachi, Tokyo, Japan) for imaging. Starch grain sizes were measured using ImageJ software, with at least 40 individual granules analyzed for size distribution.

### 2.7. Gel Permeation Chromatography Analysis

Purified rice starch (as described in Section 2.6) was enzymatically debranched using isoamylase (EC 3.2.1.68, E-ISAMY, Megazyme, Bray, County Wicklow, Ireland), dissolved in dimethyl sulfoxide (DMSO, Merck KGaA, Darmstadt, Germany), and analyzed for relative molecular weight distribution via gel permeation chromatography (GPC) using a PL-GPC 220 integrated GPC system (Agilent Technologies, Lexington, MA, USA). The resulting GPC chromatograms resolved three distinct fractions, corresponding to A and B_1_ chains of amylopectin (Ap1), long B chains of amylopectin (Ap2), and amylose (Am), respectively.

### 2.8. Total RNA Extraction and Quantitative Real-Time PCR Analysis

Total RNA was extracted from developing rice caryopses at 5, 10, 15, 20, and 25 DAF using the FastPure Universal Plant Total RNA Isolation Kit (Vazyme, Nanjing, Jiangsu, China). First-strand cDNA synthesis was performed using the HiScript III RT SuperMix for qPCR (+gDNA wiper) Kit (Vazyme, China). Quantitative real-time PCR (RT-qPCR) was conducted on a CFX Connect Real-Time PCR Detection System (Bio-Rad, Hercules, CA, USA) using ChamQ Universal SYBR qPCR Master Mix (Vazyme). The *Actin01* gene was used as an internal reference for normalization, and all experiments were performed with three biological replicates. The primers used for RT-qPCR are listed in Table S1.

### 2.9. Western Blot and Enzyme Activity Assay

Mature rice flour was weighed and suspended in total protein extraction buffer (125 mM Tris-HCl (pH 6.8), 4 M urea, 4% SDS, and 5% β-mercaptoethanol] at 37 °C for 3 h (h) at a ratio of 1:15 (1 mg flour to 15 µL buffer). The extracted total proteins were mixed with 5× protein loading buffer, denatured at 99 °C for 10 min, separated via SDS-PAGE, and subsequently transferred onto a PVDF membrane. GBSSI accumulation was examined using a specific GBSSI antibody, while SDS-PAGE gels were stained with Coomassie Brilliant Blue as a loading control [58]. GBSSI enzyme activity was assessed using a GBSSI enzyme activity assay kit (Keming, Suzhou, Jiangsu, China) with developing rice caryopses collected at 10 DAF.

### 2.10. Transient Assay of Promoter Activity

The 2.7 Kb promoter sequences of S7-edited lines and their WT counterparts were amplified and cloned into the pGreenII 0800-LUC vector. The resulting reporter constructs were introduced into *Agrobacterium tumefaciens* and subsequently infiltrated into *Nicotiana benthamiana* leaves. Firefly luciferase (LUC) and Renilla luciferase (REN) activities were measured using a Dual Luciferase Reporter Assay Kit (Vazyme). Promoter activity was quantified as the LUC/REN ratio. Three plants at the same developmental stage were used for each assay, with all experiments conducted in triplicate.

### 2.11. Full-Length Transcriptome Analysis

Full-length transcriptome sequencing of rice caryopses collected 10 DAF from both WT and S7-edited lines was performed and analyzed by Biomarker Technology Company (Beijing, China) using the PromethION platform (Oxford Nanopore Technologies, Oxford, UK).

### 2.12. Statistical Analysis

All data are presented as means ± standard deviations (SD). A one-way analysis of variance (ANOVA) was conducted using GraphPad Prism 9.3.1 software to assess statistical significance, with *p* < 0.05 (*) and *p* < 0.01 (**) indicating significant differences.

## 3. Results

### 3.1. Creation of a Novel Wx Allele (Wx^b-d25^) via Promoter Editing

**Figure 1:**
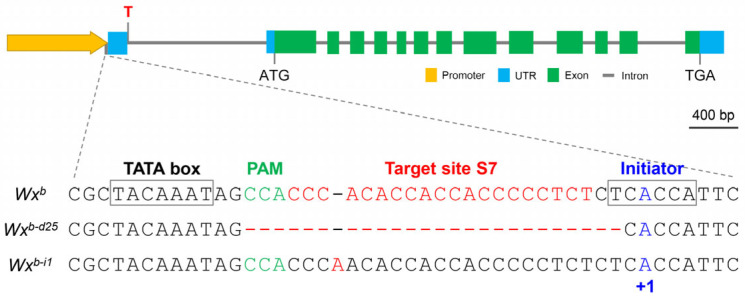
Schematic representation of sequence alterations at the S7 target site within the *Wx* promoter in the wild-type (*Wx^b^*) and the edited lines (*Wx^b-d25^* and *Wx^b-i1^*). The TATA box, PAM site, target region (S7), and transcriptional initiator are highlighted. The overall gene structure, including promoter, untranslated region (UTR), exons, introns, and termination codon (TGA), is indicated for reference.

To develop novel *Wx* alleles with enhanced regulatory properties, we generated a series of S7-edited lines, building on our previous findings that editing the S7 site within the *Wx* gene promoter significantly regulates *Wx* gene expression and AC in the rice endosperm. Screening for mutation types in the regenerated edited lines led to the identification of a novel *Wx* allele, *Wx^b-d25^*, characterized by a 25 bp deletion (−26 to −2) near the S7 site (Figure 1). The *Wx^b-d25^* allele retained the integrity of the TATA box (−35 to −29) located upstream of the S7 site but partially disrupted the transcription initiator (−2 to +4) of the *Wx* gene. To further investigate the genetic effects of *Wx^b-d25^* and evaluate its potential for breeding applications, we conducted a comprehensive analysis comparing *Wx^b-d25^* with its recipient parent, Nipponbare (*Wx^b^*), and the previously reported *Wx^b-i1^* allele, which harbors an adenine insertion [44].

We first measured the agronomic traits of the candidate lines. The results showed that, in comparison to the WT, both *Wx^b-d25^* and *Wx^b-i1^* exhibited no significant alterations in key vegetative and reproductive traits (Figure S1). A detailed analysis of agronomic data revealed that, aside from a modest reduction in grain length and 1000-grain weight, the edited lines were largely comparable to the WT (Table S2). These findings corroborate our previous report and reinforce the understanding that the *Wx* gene predominantly governs amylose synthesis in the rice endosperm [44].

### 3.2. Wx^b-d25^ Allele Synergistically Improves Amylose Content and Grain Transparency in Rice

**Figure 2:**
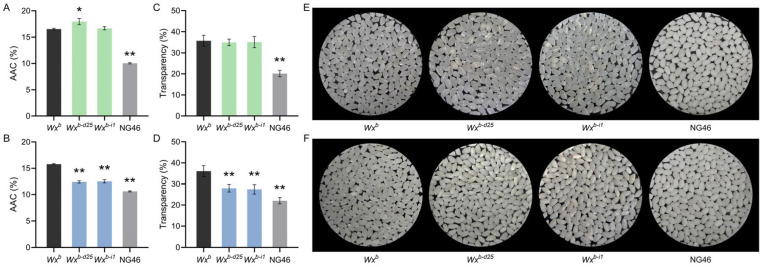
Apparent amylose content, transparency, and appearance of polished rice grains from *Wx* promoter-edited lines and the wild-type. (**A**,**C**,**E**) Data from rice grains grown under summer conditions in Yangzhou. (**B**,**D**,**F**) Data from rice grown under winter conditions in Lingshui. (**A**,**B**) Apparent amylose content (AAC); (**C**,**D**) Grain transparency values; (**E**,**F**) Representative images of polished rice grains. “*” and “**” indicate statistically significant differences compared to the wild-type at *p* < 0.05 and *p* < 0.01, respectively. *n* = 3 for AAC measurements (**A**,**B**) and transparency values (**C**,**D**); *n* = 40 for grain transparency imaging (**E**,**F**).

To elucidate the regulatory effect of the *Wx^b-d25^* allele on rice AC, and considering our previous findings that the S7 site of the *Wx* promoter influences *Wx* gene expression and AC in a temperature-dependent manner, we measured the AAC of rice grains harvested under summer planting conditions in Yangzhou and winter conditions in Lingshui. The average summer temperature in Yangzhou is approximately 2.5–3.5 °C higher than the winter temperature in Lingshui. Under the warmer summer conditions in Yangzhou, the AAC of *Wx^b-d25^* rice significantly increased from 16.5% in the WT to 18.0%, whereas *Wx^b-i1^* showed no significant change (Figure 2A). In contrast, under the cooler winter conditions in Lingshui, the AAC of both *Wx^b-d25^* and *Wx^b-i1^* was significantly reduced, from 15.8% in the WT to 12.4% and 12.5%, respectively, reaching levels characteristic of high-quality soft rice (Figure 2B). These results indicate that the *Wx^b-d25^* allele effectively modulates rice AC, but its regulatory effect, similarly to other S7-edited *Wx* alleles, is temperature-dependent.

AC is also a key determinant of rice grain transparency. To assess the impact of the *Wx^b-d25^* allele on grain transparency following AC modification, we examined the grain appearance of milled rice under both planting conditions and compared it with the high-quality soft rice cultivar NG46, which carries the *Wx^mp^* allele. Under the warmer summer conditions in Yangzhou, both *Wx^b-d25^* and *Wx^b-i1^* exhibited a transparent grain phenotype, consistent with their respective AAC profiles, which showed either no significant change or a notable increase (Figure 2C,D). In contrast, under the cooler winter conditions in Lingshui, the transparency of *Wx^b-d25^* and *Wx^b-i1^* grains was significantly reduced, aligning with their low AC phenotypes. However, in terms of measured transparency values, the polished rice from both lines exhibited significantly greater transparency than the high-quality soft rice control NG46 (Figure 2E,F). These results indicate that the *Wx^b-d25^* allele simultaneously regulates both AC and grain transparency, offering a promising strategy to enhance both ECQ and AQ in rice.

### 3.3. Wx^b-d25^ Allele Enhances Rice Grain Quality

**Figure 3:**
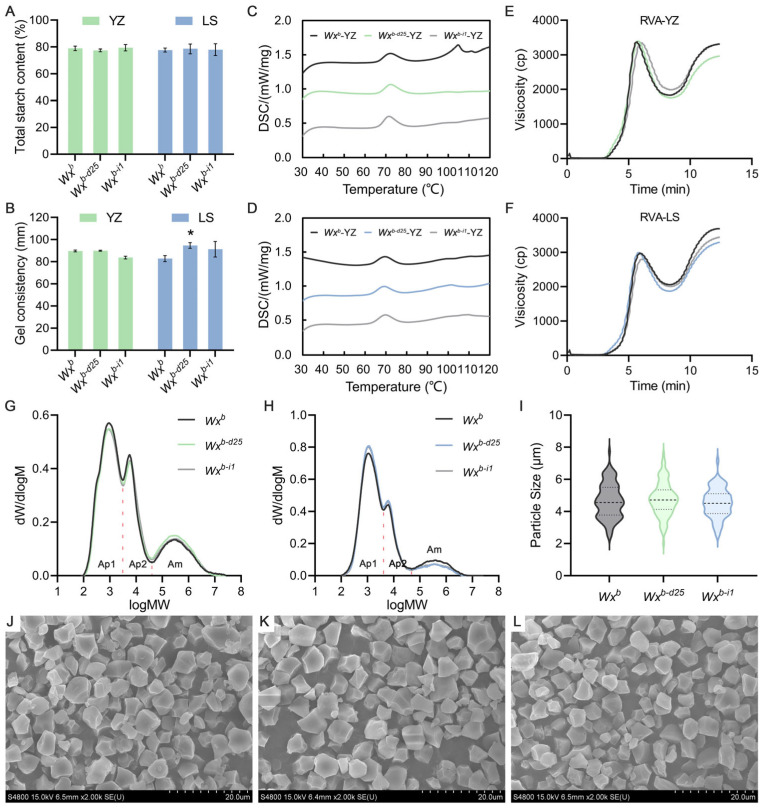
Physicochemical properties of rice grains from *Wx* promoter-edited lines and the wild-type. (**A**) Total starch content. (**B**) Gel consistency. (**C**,**D**) Differential scanning calorimetry (DSC) curves showing gelatinization temperatures of rice flour from Yangzhou (**C**) and Lingshui (**D**). (**E**,**F**) Rapid visco analyzer (RVA) profiles of rice flours from Yangzhou (**E**) and Lingshui (**F**). (**G**,**H**) Molecular weight distribution of debranched starch from grains grown in Yangzhou (G) and Lingshui (**H**). (**I**) Starch granule particle size from rice grains grown in Yangzhou. (**J**,**L**) Scanning electron microscopy (SEM) images illustrating starch granule morphology of *Wx^b^* (**J**), *Wx^b-d25^* (**K**), and *Wx^b-i1^* (**L**). YZ, Yangzhou; LS, Lingshui. An asterisk indicates a statistically significant difference at *p* < 0.05; *n* = 3.

To clarify the specific effects of *Wx^b-d25^* and *Wx^b-i1^* on rice grain quality, we assessed key physicochemical parameters, including TSC, GC, GT, and RVA profiles. Under summer planting conditions in Yangzhou, *Wx^b-d25^* and *Wx^b-i1^* exhibited no significant differences in TSC, GC, and GT compared to the WT control. Similarly, under winter planting conditions in Lingshui, both TSC and GT remained unchanged; however, GC length increased slightly, with *Wx^b-d25^* showing a statistically significant increase (Figure 3A–D, Table S3). The RVA profile of *Wx^b-i1^* rice flour from Yangzhou closely resembled that of the WT, except for a significant increase in PeT. In contrast, under winter planting conditions in Lingshui, *Wx^b-i1^* displayed a general decline in its RVA profile, with significant reductions in PKV, BDV, CPV, and SBV, while PeT was significantly increased. These changes were consistent with its significantly lower AC. Unlike *Wx^b-i1^*, *Wx^b-d25^* exhibited distinct RVA profile modifications despite differing trends and magnitudes of AC variation under the two planting conditions. In both environments, *Wx^b-d25^* showed an overall reduction in its RVA profile, with significant decreases in HPV, CPV, SBV, and PaT, while BDV was significantly increased (Figure 3E,F, Table 1). Notably, the combination of significantly increased BDV and significantly decreased SBV is consistent with the previously reported RVA characteristics of premium-tasting rice [59,60,61], indicating that *Wx^b-d25^* contributes to ECQ enhancement across different planting conditions.

In addition, we extracted starch from the tested rice grains and conducted a detailed analysis of its fine structure. GPC and SEM results revealed that the increase, stability, or reduction in AAC in *Wx^b-d25^* and *Wx^b-i1^* under different planting conditions correspondingly altered the proportion of amylopectin fractions with varying molecular weights (Figure 3G,H). However, these changes had no significant impact on the morphology or size of starch granules (Figure 3I–L). This result aligns with the previously observed AAC trends and is consistent with existing reports on the *Wx* gene’s role in starch composition.

### 3.4. Expression and Promoter Activity Analysis of Wx^b-d25^

**Figure 4:**
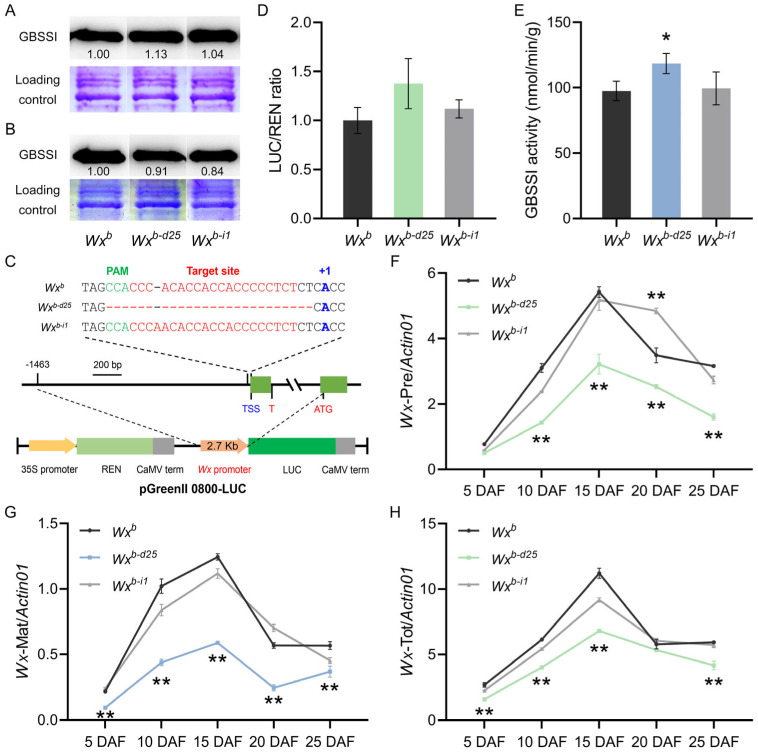
Expression and activity analyses of *Wx* promoter-edited lines and wild-type. (**A**,**B**) GBSSI protein accumulation in grains from each line grown in Yangzhou (**A**) and Lingshui (**B**). (**C**) Schematic representation of the pGreenII 0800-LUC vector construction. (**D**) Transient assay of *Wx* allele promoter activity. (**E**) GBSSI activity in developing rice caryopses at 10 DAF under Yangzhou field conditions. (**F**–**H**) Relative expression levels of *Wx* gene precursor mRNA (**F**), mature mRNA (**G**), and total mRNA (**H**) in developing caryopses grown in Yangzhou. Asterisks (* and **) indicate statistically significant differences at *p* < 0.05 and *p* < 0.01, respectively (*n* = 3).

To clarify the impact of promoter editing on *Wx* gene transcription and translation, we first conducted Western blot analysis to analyze the expression of GBSSI protein in mature grains under two distinct planting conditions. Under summer planting conditions in Yangzhou, the accumulation of GBSSI protein in *Wx^b-d25^* exhibited a significant increase, whereas *Wx^b-i1^* showed no significant change (Figure 4A). Conversely, under winter planting conditions in Lingshui, GBSSI protein accumulation was significantly reduced in both *Wx^b-d25^* and *Wx^b-i1^* (Figure 4B), aligning with their previously observed AC phenotypes. To further validate promoter activity in vitro, we employed a dual-luciferase reporter gene assay to assess transient expression levels of *Wx^b-d25^* and *Wx^b-i1^* promoters in tobacco. The results revealed an upward trend in the promoter activity of *Wx^b-d25^*, though statistical significance was not achieved. Meanwhile, the promoter activity of *Wx^b-i1^* remained comparable to that of the WT (Figure 4C,D). Additionally, developing seeds at 5–25 DAF under summer planting conditions in Yangzhou were analyzed for GBSSI enzyme activity and *Wx* gene expression. Enzyme activity assays showed a significant increase in GBSSI enzyme activity and *Wx* gene expression. Enzyme activity assays indicated a significant increase in GBSSI activity in 10 DAF in *Wx^b-d25^*, while *Wx^b-i1^* displayed no discernible change (Figure 4E). Given previous studies noting that the G/T mutation at the first base of intron 1 in the *Wx^b^* allele disrupts normal splicing by causing intron 1 retention and a significant reduction in splicing efficiency [58], we employed RT-qPCR with three primer pairs to investigate *Wx* gene expression in developing grains from 5 to 25 DAF. With a few exceptions, *Wx^b-i1^* exhibited no significant differences in precursor mRNA (*Wx*-Pre), mature mRNA (*Wx*-Mat), or total mRNA (*Wx*-Tot) expression compared to WT. This was consistent with the absence of significant changes in its AC, promoter activity, GBSSI enzyme activity, and protein expression. However, in *Wx^b-d25^*, the expression of all three transcript variants was significantly reduced, contradicting the significant increases observed in AC, promoter activity, and GBSSI protein accumulation (Figure 4F–H). Given that the 25 bp deletion in the core promoter region of *Wx^b-d25^* likely disrupted the transcriptional initiator of the *Wx* gene, we speculate that this discrepancy between phenotype and *Wx* gene transcriptional levels may stem from alterations in mRNA splicing mechanisms.

### 3.5. Full-Length Transcriptome Sequencing Reveals a Novel Transcription Mechanism in Wx^b-d25^

**Figure 5:**
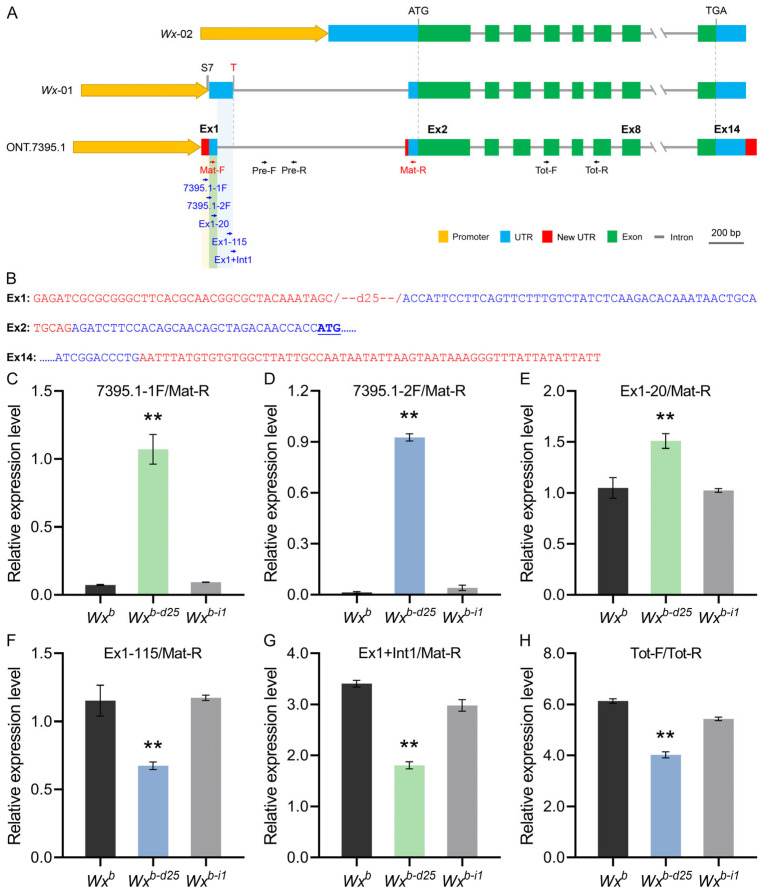
Transcript identification and validation of *Wx^b-d25^* allele. (**A**) Schematic illustration of the three transcripts derived from the *Wx^b-d25^* allele. (**B**) Sequence changes in exons 1, 2, and 14 of the ONT.7395.1 transcript. Text in red and blue represents altered and unchanged sequences relative to *Wx*-01, respectively. (**C**–**H**) Relative expression levels measured with primer sets 7395.1-1F/Mat-R (**C**), 7395.1-2F/Mat-R (**D**), Ex1-20/Mat-R (**E**), Ex1-115/Mat-R (**F**), Ex1+Int1/Mat-R (**G**), and 7395.1-1F/Mat-R (**H**). The primer locations are indicated in (**A**). Double asterisks (**) indicate statistically significant differences at *p* < 0.01 (*n* = 3).

To verify this supposition, we utilized Nanopore third-generation full-length transcriptome sequencing to investigate *Wx* gene transcripts in *Wx^b-d25^*, *Wx^b-i1^,* and their corresponding WT. Data analyses revealed that, in addition to the two canonical transcripts Os06t0133000-01 (hereafter *Wx*-01, predominantly expressed isoform) and Os06t0133000-02 (*Wx*-02), several novel *Wx* transcripts were identified. Among these, the transcript designated ONT.7395.1 closely resembles *Wx*-01 in overall structure, retaining all 14 exons without altering the protein-coding sequence. However, it exhibits distinct modifications in the sequence and length of exon 1, exon 2, and exon 14 (Figure 5A). Specifically, exon 1 of the ONT.7395.1 transcript contains a promoter sequence (−64 bp to −26 bp) along with a partial fragment of exon 1 (+1 bp to +46 bp) from the original *Wx*-01 transcript. The absence of a 25 bp segment (−26 bp to −2 bp), which was deleted through gene editing, indicates that this transcript is uniquely associated with *Wx^b-d25^*. Meanwhile, exon 2 of ONT.7395.1 is nearly identical to that of *Wx*-01, except for a 5 bp (TGCAG) insertion derived from the terminal region of intron 1 in the original *Wx*-01 transcript, which is added at the exon’s 5′ end. It is particularly noteworthy that the sequence alterations in exon 1 and exon 2 of ONT.7395.1 effectively bypass the G/T mutation at the 5′ splicing site of intron 1 in *Wx^b^*, a mutation known to severely impair splicing efficiency. Additionally, exon 14 of ONT.7395.1 extends 60 bp beyond the original *Wx*-01 transcript (Figure 5B). These findings suggest that the 25 bp deletion in *Wx^b-d25^* alters the splicing pattern of the *Wx* gene, leading to the generation of a novel transcript. This transcript retains the full protein-coding sequence but undergoes structural modifications that may influence its stability, expression, or regulatory interactions.

To further validate the authenticity and expression levels of the ONT.7395.1 transcript and investigate the transcriptional mechanism of *Wx^b-d25^*, we designed multiple RT-qPCR primers targeting distinct *Wx* transcripts. This was particularly necessary given that the original Mat-F primers (+4 bp to +29 bp), used for detecting mature *Wx* mRNA expression, are located near the transcription initiator of the *Wx* gene. Among the designed primers, 7395.1-1F and 7395.1-2F primers specifically detected the ONT.7395.1 transcript, while the Ex1-20 primer identified both the *Wx*-01 and ONT.7395.1 transcripts. The Ex1-115 primer was selective for *Wx*-01, and the Ex1 + Int1 primer specifically detected the pre-mRNA containing unspliced intron 1 of *Wx*-01. Expression analysis of developing grains at 10 DAF confirmed that the ONT.7395.1 transcript was uniquely present and actively expressed in *Wx^b-d25^* (Figure 5C,D). Additionally, expression levels of precursor mRNA, mature mRNA, and total mRNA of the *Wx*-01 transcript were significantly reduced compared to the WT, indicating that although *Wx*-01 remained transcribed, its transcription was significantly reduced by approximately 50% (Figure 5F–H).

In terms of specific expression levels, the ONT.7395.1 transcript in developing grains of *Wx^b-d25^* at 10 DAF exhibited an expression level of approximately 0.9–1.1 relative to the internal reference *Actin01* gene (Figure 5C,D). Despite this relatively low expression, ONT.7395.1 was fully translated into functional GBSSI protein without being affected by compromised splicing efficiency. Meanwhile, the *Wx*-01 transcript in *Wx^b-d25^* exhibited an overall expression level of 2.5 (sum of precursor mRNA and mature mRNA) relative to the internal reference *Actin01* gene (Figure 5F–G), representing approximately 55% of WT (*Wx^b^*) expression. However, due to the G/T variation at the intron 1 splice site, only about 0.7 of this fraction was successfully processed into mature *Wx* mRNA (Figure 5F). The combined expression of these two transcripts resulted in a significant increase in the mature *Wx* mRNA encoding GBSSI protein in *Wx^b-d25^* compared to the WT (Figure 5E). This, in turn, led to the elevated AC and physicochemical changes of starch in *Wx^b-d25^* under summer planting conditions.

## 4. Discussion

### 4.1. Development of an Elite Wx Allele

AC is a crucial factor in determining rice grain quality, with the *Wx* gene serving as the primary genetic regulator. Modulating the expression and activity of the *Wx* gene and its encoded GBSSI protein to optimize endosperm amylose synthesis is a critical approach to improving rice grain quality. Existing strategies for regulating *Wx* and GBSSI expression primarily include (1) utilization of superior *Wx* alleles with low AC, such as *Wx^b^* (AC~16%), *Wx^mw^*/*Wx^la^* (AC~13%), and *Wx^mp^* (AC~10.5%) [3,14,21]; (2) transcriptional regulation of *Wx* through specific transcription factors; (3) regulation of *Wx* splicing efficiency by *Dull* genes and QTLs; (4) indirect modification of AC via other starch synthesis-related genes, particularly those involved in amylopectin synthesis (e.g., *SBEIIb*, *SSIIIa*) and non-enzymatic proteins that facilitate GBSSI localization to the starch granule surface (e.g., *OsGBP*, *OsLESV*, *OsESV1*) [62,63,64,65,66]; and (5) regulation of genes that influence starch synthesis indirectly, including those controlling floury endosperm formation [67,68], chalkiness [69], hormone synthesis and transport [70], and grain filling [71]. However, most of these genetic modifications not only alter AC but also lead to compromised yield and some undesirable quality traits, such as floury endosperm and high chalkiness. Consequently, despite the extensive identification of genes directly or indirectly involved in amylose biosynthesis, few have been proven suitable for rice breeding programs. Instead, the incorporation of superior alleles of key starch synthesis genes, particularly *Wx,* remains the predominant strategy for breeding high-quality rice varieties [72]. For rice consumers in East Asian regions such as China and Japan, rice with low AC is widely popular due to its lower hardness and higher stickiness [12]. In current low-AC rice breeding programs, the superior *Wx* alleles primarily utilized are *Wx^b^* (AC around 16%) and *Wx^mp^* (AC about 10.5%), respectively aligning with conventional rice cultivars and soft rice varieties [3]. Rice carrying the *Wx^b^* allele generally exhibits inferior ECQ compared to soft rice varieties carrying the *Wx^mp^* allele, which have even lower AC [52]. However, grains carrying the *Wx^b^* allele maintain high transparency. Although soft rice varieties carrying *Wx^mp^* are more favored by Chinese consumers, their grains have low transparency and poor AQ due to the extremely low AC [14,27,52].

Grain transparency is a key component of rice AQ and is positively correlated with AC. High transparency forms the foundation of excellent AQ [52]. AC and RVA are key physicochemical indicators for assessing rice ECQ [73]. Early studies found that RVA profiles are significantly correlated with rice AC and texture (hardness and stickiness). Rice varieties with higher AC typically exhibit lower BDV and higher SBV [59]. For rice texture, changes in rice hardness and stickiness are significantly correlated with changes in BDV and SBV in the RVA profile. In rice production, high-quality varieties with good taste are generally recognized to have larger BDV and smaller SBV. Conversely, varieties with poor taste often have smaller BDV and larger SBV [60,61].

In this study, we developed a novel *Wx^b-d25^* allele by further editing the S7 site in the core promoter region of the *Wx* gene, as previously reported [44]. Compared with the wild type (*Wx^b^*) and the reported *Wx^b-i1^* control, *Wx^b-d25^* showed no significant changes in agronomic traits, consistent with other reports on *Wx* gene editing [45]. Physicochemical quality analysis revealed that under summer planting conditions in Yangzhou, the AC of *Wx^b-d25^* rice increased slightly compared with the wild type, while its grain transparency remained high. In contrast, under winter planting conditions in Lingshui, both the AC and transparency of *Wx^b-d25^* rice significantly decreased. The AC approached that of the high-ECQ soft rice control NG46, but the transparency remained significantly higher than that of the latter. Further RVA analysis showed that under both planting conditions, *Wx^b-d25^* rice exhibited characteristics similar to those of high-ECQ rice, with a significant increase in BDV and a significant decrease in SBV, suggesting that its rice texture and final ECQ might be improved under both conditions. We speculate that under Yangzhou summer planting conditions, the significant increase in AC of *Wx^b-d25^* rice, yet exhibiting RVA characteristics similar to those of elite low-AC rice, might be due to the relatively small increase in AC combined with significant decreases in HPV, CPV, and PaT. These results indicate that *Wx^b-d25^* has the potential to enhance rice ECQ by optimizing texture and maintaining high transparency under conventional summer conditions, building on the high AQ of *Wx^b^* rice. Under slightly lower winter planting conditions, it can moderately reduce rice AC and optimize rice texture, thereby bringing its ECQ closer to that of high-quality soft rice carrying the *Wx^mp^* allele while maintaining significantly higher grain transparency than the latter. Therefore, despite significant differences in AC variation under different planting conditions, *Wx^b-d25^* has the potential to simultaneously improve both rice ECQ and AQ. Among these, the enhancement of rice AQ through grain transparency by *Wx^b-d25^* is readily observable. However, the impact of *Wx^b-d25^* on rice ECQ, given the complexity of its definition and the diverse dietary preferences of consumers, still requires further validation, such as through human sensory evaluation.

### 4.2. Revelation of a Novel Transcriptional Pattern of the Wx Gene

The advancement of gene editing technologies has facilitated the creation of superior *Wx* alleles devoid of foreign T-DNA insertions, making them highly applicable for rice breeding [74]. Currently, various gene editing approaches, including *Wx* gene knockout, promoter editing, upstream open reading frame (uORF) modification, single-base editing, and splice-site editing, have been successfully implemented in rice [13,44,45,48,75,76,77,78,79]. However, these studies have primarily focused on modifying key regulatory sites previously identified as influencing *Wx* gene expression, GBSSI activity, and splicing efficiency. While promoter editing has revealed novel regulatory elements within the *Wx* promoter, it has not been shown to induce significant alterations in the transcriptional pattern of the *Wx* gene [45,79].

In this study, the S7 site was identified within the core promoter region of the *Wx* gene. The 25-base deletion in this region partially disrupted the transcription initiator, not only affecting *Wx* gene expression but also altering its transcriptional pattern, leading to the emergence of a novel transcript, ONT.7395.1. Interestingly, despite substantial sequence modifications in ONT.7395.1, Western blot analysis confirmed that the GBSSI protein it encodes remained unchanged. While the original *Wx*-01 transcript persisted in *Wx^b-d25^*, its expression was significantly reduced due to the partial disruption of the transcription initiator. In contrast, ONT.7395.1, though expressed at a lower level, retained full splicing efficiency and was completely processed into mature *Wx* mRNA. The co-existence of both the original and novel transcripts resulted in an overall increase in *Wx* gene expression, leading to a higher GBSSI protein accumulation and subsequent changes in AC and physicochemical properties in *Wx^b-d25^* rice under Yangzhou summer planting conditions. These results not only uncover a novel transcriptional pattern of the *Wx* gene but also provide new insights into promoter editing as a tool for modulating gene expression. However, since the *Wx^b-d25^* allele only partially disrupts the transcription initiator, further research is needed to explore the effects of its complete disruption on *Wx* gene transcription.

The core promoter, a minimal DNA sequence essential for initiating transcription in eukaryotes, serves as a platform for the RNA polymerase system to recognize and bind to the gene promoter [80,81]. Typical eukaryotic gene core promoters usually contain *cis*-acting elements recognized and bound by general transcription factors (GTFs), such as the initiator and the TATA box [82,83,84,85]. In *Wx^b-d25^*, the deletion of 25 bases did not disrupt the TATA box on the *Wx* core promoter but partially disrupted the transcription initiator and may have affected other unknown *cis*-acting elements (Figure 1). This likely altered the binding efficiency of one or more GTFs, thereby changing the transcription of the downstream *Wx* gene. The AC phenotype of *Wx^b-d25^* is highly temperature-sensitive, showing opposite trends in AC changes under Yangzhou and Lingshui planting conditions, yet maintaining an AC above 12%. This suggests that while GTFs can still recognize and bind to the modified *Wx* core promoter, their recognition or binding to the edited region is significantly less thermally stable. This highlights the need for further investigation into their regulatory mechanisms under varying environmental conditions. Due to the absence of RNA samples from Lingshui, we are currently unable to determine the specific temperature response of the two transcripts.

## 5. Conclusions

In conclusion, we successfully developed the novel *Wx^b-d25^* allele by editing the core promoter of the *Wx* gene. Compared with the wild-type control and *Wx^b-i1^*, *Wx^b-d25^* exhibited no significant changes in agronomic traits. Through comprehensive physicochemical quality analyses, we demonstrated that this allele exhibits a temperature-responsive pattern of AC regulation under different planting conditions and has the potential to synergistically enhance both the ECQ and AQ of rice by modulating AC, grain transparency, and texture. Moreover, we uncovered a unique transcriptional pattern in *Wx^b-d25^*, characterized by the co-regulation of *Wx* gene expression and rice AC through dual transcripts (*Wx*-01 and ONT.7395.1) under different planting conditions. These findings not only provide valuable insights for leveraging gene editing technology to precisely regulate target gene expression via promoter editing but also lay a solid theoretical foundation for using the *Wx^b-d25^* allele to optimize endosperm starch composition and structure, ultimately improving rice grain quality.

Building on these research findings, the *Wx^b-d25^* allele can be introduced into cultivated rice varieties to systematically evaluate its potential for breeding applications. Additionally, further editing of the core promoter of the *Wx* gene, including the creation of new *Wx* alleles with complete deletion of the initiator, can be carried out. This approach will not only aid in further elucidating the transcriptional regulation mechanisms of the *Wx* gene but also address the increasing demands for variations in rice AC and physicochemical quality driven by consumers’ diverse dietary preferences.

## Figures and Tables

**Table 1:** Rapid visco analyzer (RVA) parameters of rice flour.

Samples	PKV (cP)	HPV (cP)	BDV (cP)	CPV (cP)	SBV (cP)	PeT (Min)	PaT (°C)
*Wx^b^*-YZ	3430.2 ± 55.7	1965.1 ± 127.2	1468.5 ± 71.5	3430.7 ± 117.2	3.9 ± 62.2	5.7 ± 0.1	79.7 ± 0.8
*Wx^b-d25^*-YZ	3413.8 ± 14.5	1747.6 ± 10.0 *	1669.6 ± 22.6 *	3077.4 ± 107.2 **	−331.1 ± 94.2 **	5.8 ± 0.0	76.6 ± 0.0 **
*Wx^b-i1^*-YZ	3423.2 ± 72.2	1987.2 ± 7.4	1439.4 ± 77.2	3298.7 ± 19.6	−121.2 ± 90.5	6.0 ± 0.0 **	78.1 ± 0.8
*Wx^b^*-LS	2978.4 ± 6.2	2051.0 ± 11.3	930.7 ± 13.6	3725.4 ± 33.9	750.4 ± 31.9	6.0 ± 0.0	87.7 ± 0.1
*Wx^b-d25^*-LS	2944.2 ± 48.6	1859.0 ± 10.4 *	1088.5 ± 39.7 *	3278.3 ± 16.2 **	337.4 ± 33.9 **	5.8 ± 0.0 **	81.4 ± 3.1 **
*Wx^b-i1^*-LS	2495.3 ± 242.1 **	2003.6 ± 141.0	795.0 ± 101.0 **	3437.8 ± 152.5 *	645.8 ± 90.2 *	6.1 ± 0.0 **	88.6 ± 0.8

Note: PKV, peak viscosity; HPV, hot paste viscosity; BDV, breakdown value; CPV, cool paste viscosity; SBV, setback value; PeT, peak time; PaT, pasting temperature; YZ, Yangzhou; LS, Lingshui. Data are presented as means ± standard deviations (SD) (*n* = 3). A single asterisk (*) and double asterisk (**) indicate statistically significant differences at *p* < 0.05 and *p* < 0.01, respectively.

## Data Availability

The original contributions presented in the study are included in the article/Supplementary Material, further inquiries can be directed to the corresponding author.

## Supplementary Materials

The following supporting information can be downloaded at: https://www.mdpi.com/article/10.3390/foods14081330/s1, Figure S1: Morphology of rice plants and grains from *Wx^b-d25^* and the wild-type; Table S1: Primers used in this study; Table S2: Main agronomic traits of *Wx* promoter-edited lines and the wild-type; Table S3: DSC parameters of rice flour; Table S4: The physiological parameters, methods, reagents and equipment involved in this study [3,5,9,27,52,56,58].
