# Supplementary material for: Creating a Superior Wx Allele with Temperature-Responsive Amylose Regulation and a Novel Transcriptional Pattern in Rice via CRISPR/Cas9-Mediated Promoter Editing
Source: Foods. 2025 Apr 11;14(8):1330. doi: 10.3390/foods14081330 (PMC12026567; doi:10.3390/foods14081330)
Supplement: Supplementary file 1 [file foods-14-01330-s001.zip › foods-3551439-supplementary.pdf]

Supplementary Materials

Figure S1. Morphology of rice plants and grains from *Wx*$^{b\text{-}d25}$ and the wild-type.

Table S1. Primers used in this study.

Table S2. Main agronomic traits of *Wx* promoter-edited lines and the wild-type.

Table S3. DSC parameters of rice flour.

Table S4. The physiological parameters, methods, reagents and equipment involved in this study.

Supplementary Figures

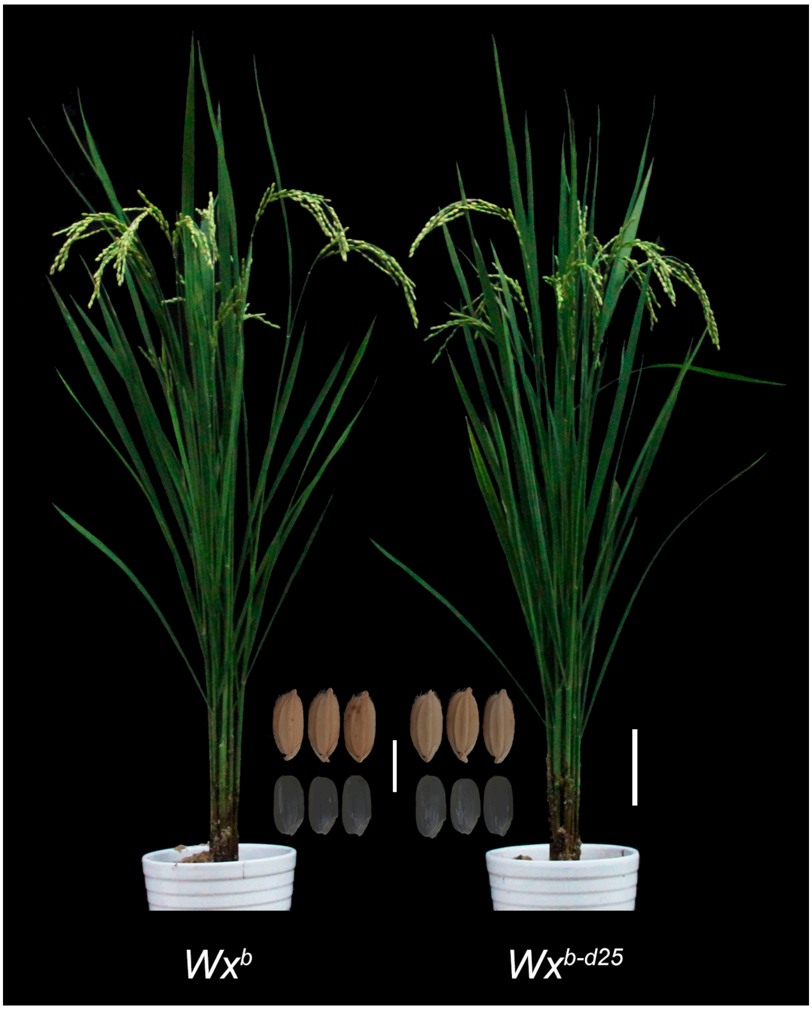

Figure S1. Morphology of rice plants and grains from *Wx^b-d25* and the wild-type.

Supplementary Tables

Table S1. Primers used in this study.

Name of primer	Primer sequences (5'-3')	Purpose
S7-F	ggcaAGAGGGGGTGGTGGTGTGGG	Target site primers
S7-R	aaacCCCACACCACCACCCCCTCT	
Hyg-F	GCTTCTGCGGGCGATTTGTGT	*HPT* gene identification
Hyg-R	GGTCGCGGAGGCTATGGATGC	
S7 Test-F	CGGGTAAAATGTGTTGCGG	Detection of mutation on *Wx* promoter
S7 Test-R	ACTTGCAGATGTTCTTCCTGATGA	
Wxpro 0800-F	ctatagggcgaattgggtaccACCGGTTTTAATAGTTGAGAGACCC	Amplification of *Wx* promoter
Wxpro 0800-R	cgctctagaactagtggatccGGTGGTTGTCTAGCTGTTGCTGT	
Wx Pre-F	ATCTTTCATTGCTCGTTTTTCCTTA	qRT-PCR
Wx Pre-R	GCCTAACCAAACATAACGAACGA	
Wx Mat-F	ATTCCTTCAGTTCTTTGTCTATCTCA	
Wx Mat -R	ATGGTGGTTGTCTAGCTGTTGC	
Wx Tot-F	CGTCATTCCTGGAGAAGGTTTG	
Wx Tot-R	CAGACGAACACAACATCCTCACC	
Ex1-20	GTCTATCTCAAGACACAAATAACTGCAG	
Ex1-115	GTTCATCAGGAAGAACATCTGCAA	
7395.1-1F	CGCAACGGCGCTACAAATAG	
7395.1-2F	GCGCTACAAATAGCACCATTC	
Ex1+Int1	AGGAAGAACATCTGCAAGTTATACA	
Actin01-F	CCAAGGCCAATCGTGAGAAGA	
Actin01-R	AATCAGTGAGATCACGCCCAG	

Table S2. Main agronomic traits of *Wx* promoter-edited lines and the wild-type.

Samples	Plant height (cm)	Tiller number	Seed setting rate (%)	Grain length (mm)	Grain width (mm)	Grain thickness (mm)	1000 grain weight (g)
Wx^{b}	94.8±1.8	7.1±1.9	93.01±4.02	7.41±0.06	3.45±0.13	2.18±0.06	25.40±0.13
$Wx^{b\text{-}d25}$	93.8±2.0	7.8±1.4	92.36±4.15	7.30±0.04*	3.39±0.06	2.17±0.08	24.62±0.20*
$Wx^{b\text{-}i1}$	95.0±1.8	7.7±1.1	90.79±3.42	7.23±0.06**	3.33±0.11	2.16±0.06	23.28±0.08**

"*" and "**" indicate statistically significant differences at $P < 0.05$ and $P < 0.01$, respectively.

Table S3. DSC parameters of rice flour.

Location	Samples	△H (J/mol)	T_o (°C)	T_p (°C)	T_e (°C)
YZ	Wx^{b}	6.27±0.08	66.15±0.07	72.00±0.14	80.00±0.42
	$Wx^{b\text{-}d25}$	6.44±0.00	66.3 0±0.42	71.85±0.21	79.60±0.14
	$Wx^{b\text{-}i1}$	6.35±0.58	66.45±0.21	72.05±0.21	79.45±0.35
LS	Wx^{b}	5.75±0.22	62.70±0.10	69.40±0.10	77.10±0.10
	$Wx^{b\text{-}d25}$	5.64±0.55	63.00±0.00	69.10±0.10	76.40±0.28
	$Wx^{b\text{-}i1}$	5.730±0.31	63.60±0.10	69.80±0.10	77.00±0.10

YZ, Yangzhou; LS, Lingshui. All data are means ± standard deviations, n=3. △H, enthalpy of gelatinization; T_o, onset temperature; T_p, peak temperature; T_e, end temperature.

Table S4. The physiological parameters, methods, reagents and equipment involved in this study.

Physiological parameters	Methodology followed or equipment used	Description	Citations
Total starch content (TSC)	Total starch assay kit (Megazyme, Ireland)	Determination of starch content	[52]
Apparent amylose content (AAC)	China's national standard (GB/T 15683-2025)		
Gel consistency (GC)	China's national standard (GB/T 22294-2008)		
Gelatinization temperature (GT)	200 F3 differential scanning calorimeter (DSC, Netzsch, Germany), providing onset (T_o), peak (T_p), and end (T_e) temperatures, along with the enthalpy of gelatinization (△H)	Assessing rice eating and cooking quality	[5,9,58]
Pasting properties	Techmaster rapid visco-analyzer (RVA, Newport Scientific, Australia), providing peak viscosity (PKV), hot paste viscosity (HPV), breakdown value (BDV), cool paste viscosity (CPV), setback value (SBV), peak time (PeT), and pasting temperature (PaT)		
Grain transparency	SC-E rice appearance quality detector (Wanshen, China) and ImageJ software	Assessing rice appearance quality	[27,52,56]
Scanning electron microscopy (SEM)	Hitachi S-4800II environmental scanning electron microscope (Hitachi, Japan) and ImageJ software	Observation of starch granule morphology and measurement of their particle size.	[52]
Gel permeation chromatography (GPC)	Isoamylase (EC3.2.1.68, E-ISAMY, Megazyme) and PL-GPC 220 integrated GPC system (Agilent Technologies, USA), providing Ap1 (A and B_1 chains of amylopectin), Ap2 (long B chains of amylopectin), and Am (amylose), respectively.	Determination of the molecular weight distribution of debranched starch.	[3,52]
